# *Drosophila* Morgue Associates with SkpA and Polyubiquitin In Vivo

**DOI:** 10.1371/journal.pone.0074860

**Published:** 2013-09-30

**Authors:** Ying Zhou, Yiqin Wang, Barbara A. Schreader, John R. Nambu

**Affiliations:** 1 Department of Biology, University of Massachusetts, Amherst, Massachusetts, United States of America; 2 Department of Biological Sciences, Florida Atlantic University, Boca Raton, Florida, United States of America; University of North Carolina at Greensboro, United States of America

## Abstract

Morgue is a unique ubiquitination protein that influences programmed cell death and circadian rhythms in *Drosophila*. We have found that over-expression of wild-type Morgue results in organismal lethality. This over-expression phenotype was used as the basis for an *in vivo* functional assay to investigate the importance of the Morgue zinc finger, F box, Ubiquitin E2 Conjugase Variant (UEV) domain, and active site Glycine residue. Removal of the zinc finger or UEV domain reduced Morgue’s ability to induce lethality and enhance cell death. In contrast, lack of the F box as well as several different substitutions of the active site Glycine did not alter Morgue-induced lethality or cell death enhancement. To further characterize Morgue functions, a Flag:Morgue protein was used to isolate Morgue-associated proteins from whole adult *Drosophila*. Mass spectrometry analysis of the Morgue-associated proteins identified SkpA as well as a ubiquitin multimer. The identification of SkpA is consistent with previous *in vitro* studies and further suggests Morgue acts in an SCF-type ubiquitin E3 ligase complex. The identification of poly-ubiquitin was unexpected and this interaction had not been previously identified. The associated poly-ubiquitin was found to exhibit a Lys-48 topology, consistent with distinct functions of Morgue in proteasome-mediated protein turnover. Multiple regions of Morgue were subsequently shown to be required for poly-ubiquitin binding. Overall, Morgue is a novel multi-functional ubiquitin-binding protein.

## Introduction

The ubiquitin/proteasome pathway influences a wide range of cellular processes, including mitosis, protein processing and quality control, response to infection, and programmed cell death [1]-[3]. Modification of proteins by ubiquitin involves the actions of a conserved enzymatic cascade, generally consisting of an E1 activator, E2 conjugase, and E3 ligase. Each of these enzymes is characterized by the presence of highly conserved domains that possess distinctive functional properties. E1 activators are large multi-domain proteins that promote COOH terminal acyl-adenylation, activation, and covalent binding of mono-ubiquitin. The activated ubiquitin is subsequently transferred to an E2 conjugase. E2s are typically small proteins that contain a ∼130 amino acid conjugase domain. This domain is characterized by the presence of several residues that interact with ubiquitin and an active site Cysteine residue that forms a covalent thioester linkage to the COOH-terminal Glycine residue of ubiquitin. Interestingly, there exists a distinct class of Ubiquitin E2 Enzyme Variants (UEVs) that contain conjugase domains lacking the active site Cysteine residue; UEVs cannot covalently attach to ubiquitin. While the functions of UEVs are not well characterized, the yeast MMS2 and human TSG101 proteins can both non-covalently associate with ubiquitin monomers and share key residues important for ubiquitin association with active E2s [4]–[9]. MMS2 acts together with Ubc13 to promote Lys-63 linkage of ubiquitin on DNA repair enzymes [4], [5], [10] while TSG101 functions in the vacuolar protein sorting pathway and is essential for HIV-1 budding [11], [12]. Other UEVs may act to negatively regulate the activity of catalytically active E2s. Catalytically active E2s typically promote target protein ubiquitination by acting in conjunction with an E3 ligase. E3s can correspond to single polypeptides such as HECT domain or some RING domain proteins, or they can consist of multi-peptide complexes such as the APC, SCF, or VCB E3 ligase complex [13]–[15]. In particular, the SCF complex minimally consists of a Cullin protein, a Skp protein, an F box protein, and RING domain protein. The Cullin scaffold interacts with the Skp protein that in turn, binds the F box domain in the F box protein. The F box protein also harbors a distinct substrate-binding domain that directly associates with the target protein. The RING domain protein binds to a ubiquitin-bearing E2 that brings ubiquitin to the substrate. Structural studies have revealed the necessity for a precise arrangement and spacing of the SCF complex subunits to permit addition of ubiquitin moieties to specific substrates [16].

Substrate proteins can undergo modification by single or multiple mono-ubiquitin moieties and by addition of polyubiquitin chains. The number and stoichiometry of ubiquitin moieties added to a substrate determine the fate of the target protein [17]. For example, mono-ubiquitination may target a protein for endocytosis or a distinct location within the cell [18], [19]. Ubiquitin is typically added to Lysine residues in the substrate protein and within ubiquitin itself, there are 7 Lysine residues and the initiator Methionine that can be used as ubiquitin addition sites for formation of a polyubiquitin chain. Generation of branched polyubiquitin chains via the ubiquitin Lys48 residue targets the substrate for degradation via the 26S proteasome, whereas Lys63-branched polyubiquitin chains mark proteins for functions in DNA-damage response or NF-kB pathway. The complexity of potential ubiquitination patterns greatly increases the information content of ubiquitination modifications and subsequent downstream responses.

In addition to the canonical ubiquitination enzymes, there exist distinct types of ubiquitin-binding proteins and ubiquitin-related proteins. The former includes several classes of proteins that may bind ubiquitin monomers or polyubiquitin [20]–[22]. Ubiquitin-binding proteins include de-ubiquitinating enzymes (DUBs) that remove ubiquitin moieties from ubiquitinated proteins. This process can aid in ubiquitin recycling, repression of ubiquitin-dependent protein turnover, or activation of a ubiquitinated protein. Ubiquitin-related proteins include SUMO and Nedd8 that can also be incorporated into ubiquitin-bearing chains [23], [24]. One example of an atypical ubiquitination protein is Morgue, which may combine E2 conjugase and an E3 ligase functions. Morgue was initially identified in *Drosophila* via two cell death genetic modifier screens [25], [26]. Expression of Morgue enhances the eye cell death phenotypes associated with *irregular chiasm C-roughest* mutants as well as targeted expression of a Reaper/Grim hybrid protein. Morgue was further shown to induce caspase-mediated death of cultured insect cells and *in vitro* assays indicated that Morgue directly associates with SkpA and DIAP1. Loss-of-function *morgue* mutants exhibit a slight increase in the number of surviving embryonic midline glia as well as alterations in the normal numbers and positions of other glia and neurons [27]. In addition, Morgue has been shown to regulate the circadian response to light [28]. Overall, these genetic data suggest roles for Morgue in the differentiation and/or survival of neurons and glia, as well as circadian rhythm pathways.

The Morgue protein contains a NH_2_-terminal zinc finger, a central F box, and COOH-terminal UEV domain [29]. Morgue is phylogenetically conserved in a diverse but discrete subset of invertebrates including members of the Lophotrochozoa, Platyzoa, and Ecdysozoa superphyla [30]. In all but one examined species, the Morgue protein exhibits the same domain organization; the 20 amino acid C4 type zinc finger exhibits an essentially invariant CX_2_CX_12_CX_2_C motif; the F box typically contains five conserved Tryptophan residues; and the UEV domain contains a Glycine replacement for the active site Cysteine present in catalytically active E2s. No other UEV contains an active site Glycine. The strong conservation of this domain architecture suggests that this organization provides essential and unique capabilities to Morgue. In addition, the active site Glycine residue may also be also critical for normal Morgue function. However, despite the existence of conserved domains, the precise function of Morgue in ubiquitination processes is not yet clear. In particular, it is not established that Morgue acts as a bona fide F box protein in an SCF complex, a UEV, or a protein with distinct ubiquitination activities. It is also uncertain what distinctive functional properties may be conferred by the active site Cysteine-to-Glycine substitution.

In this study we show that Morgue over-expression can result in organismal lethality. This phenotype is not dependent upon an intact F box domain, although it is influenced by the zinc finger and UEV domain. In contrast, the ability of Morgue to enhance R/Grim-induced eye cell death is not dependent upon the zinc finger, F box, or UEV domain, as removal of each domain singly did not significantly disrupt Morgue’s ability to enhance R/Grim-induced eye cell death. Furthermore, alterations of the active site Glycine residue did not significantly alter Morgue activity, either in inducing animal lethality or enhancing eye cell death. Finally, we use an *in vivo* protein interaction assay to isolate and purify Morgue-associated proteins from adult fly tissues. Mass spectrometry analyses revealed that Morgue directly associates with SkpA and Lys48-linked ubiquitin multimers. We show that binding between Morgue and polyubiquitin is mediated through multiple regions of the Morgue protein. Thus, Morgue is a novel ubiquitin binding protein. These data provide evidence that the individual Morgue domains provide distinct, specific functions as well as act together. Further analyses of Morgue function may provide insight into novel pathways of protein ubiquitination and proteolysis.

## Materials and Methods

### Drosophila strains

The P[*daughterless*-Gal4] (P[*da*-Gal4]), P[*elav*-Gal4], and P[UAS-GFP] lines were obtained from the Bloomington *Drosophila* Stock Center (http://flystocks.bio.indiana.edu/). P[UAS-R/Grim] and P[52a-Gal4] were previously generated by the Nambu lab [26]. The P[UAS-Morgue] strain was generated by directionally cloning a full length *morgue* cDNA into the EcoRI and XhoI sites of the pUAST vector [31] pUAST-*morgue* construct DNA was purified using a plasmid purification kit (Qiagen; Valencia, CA) and used to generate *Drosophila* germline transformants (Bestgene Inc.; Chino Hills, CA). Five independent transformant lines were obtained and genetically mapped and balanced or homozygosed.

### Generation of P[UAS-Morgue] strains encoding morgue deletion, gly421x point mutant, or 3xFLAG-tagged proteins

**Figure 1:**
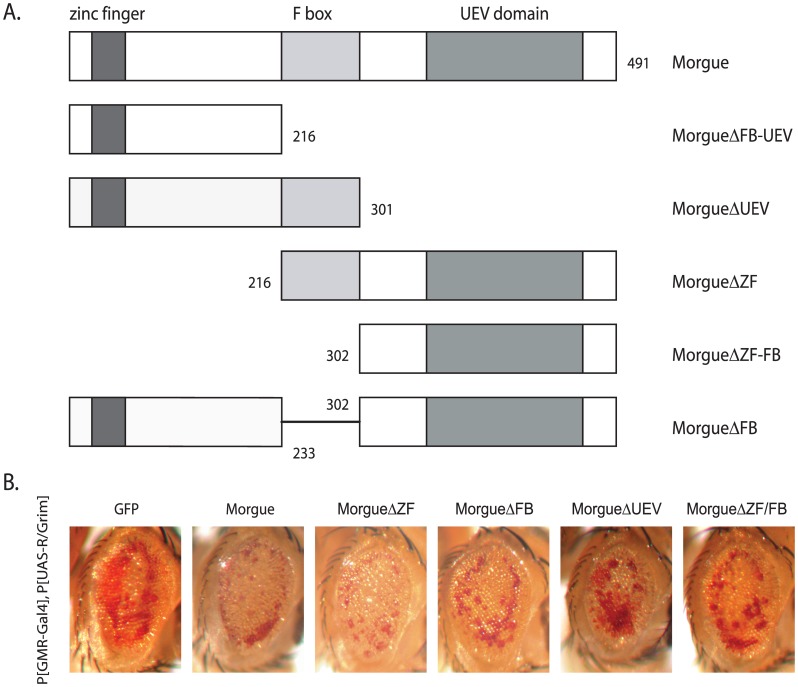
Morgue-induced in lethality is independent of the F box. **A.** Schematic representation of full length Morgue (Morgue) and Morgue deletion mutants (Morgue**Δ**). **B.** Enhancement of eye cell death by Morgue deletion mutants. Compared to GFP (P[UAS-GFP]), co-expression of Morgue (P[UAS-Morgue]) enhances the eye cell death in P[GMR-Gal4], P[UAS-R/Grim] flies. Similar enhancement is observed for co-expression of Morgue deletion mutants lacking the zinc finger (P[UAS-MorgueΔZF]), F box (P[UAS-MorgueΔZF]), UEV domain (P[UAS-MorgueΔUEV]), or zinc finger+F box (P[UAS-MorgueΔZF/FB]).

To generate Morgue Gly421 mutations, Ala, Cys, or Ser substitutions of the Gly421 residue were generated via PCR-mediated point mutation of the Morgue coding region using a full length *morgue* cDNA clone template. The Morgue deletion mutants were generated by overlap extension PCR of the *morgue* cDNA to eliminate the specific regions indicated in Figure 1. PCR products were cloned into the Eco RI and Xho I sites of the pUAST vector. The sequence fidelity of each construct was confirmed via DNA sequence analysis (Davis Sequencing; Davis CA.).

To generate the 3xFLAG Morgue constructs, a 3xFLAG sequence was amplified from the p3xFLAG-CMV vector (Sigma-Aldrich; St. Louis MO). A modified pUAST-*morgue* construct was generated using PCR-mediated point mutation to introduce a silent mutation that eliminates an internal Bgl II restriction site at residue 179 in the Morgue coding region. This modified Morgue sequence was subcloned into pUAST using the Bgl II and Xho I sites. The 3xFLAG fragment was inserted between the Eco RI and Bgl II sites (for N-terminal tagging) or Bgl II and Xba I sites (for C-terminal tagging) of the pUAST-*morgue* construct. For the 3xFLAG Morgue deletion mutants, partial Morgue coding sequences were amplified and cloned into the Eco RI and Xho I sites of the pUAST vector. In addition, a 3xFLAG tag sequence was inserted into Xho I and Xba I site (C-terminal tagging). The fidelity of each construct was verified by DNA sequence analysis (Davis Sequencing; Davis, CA). The construct DNAs were purified using a Qiagen Maxi-prep kit (Qiagen) and used to generate *Drosophila* germline transformants (Bestgene Inc.). For each construct, 6–10 transformant lines were obtained. The transformant flies were mapped and balanced or homozygosed. At least two independent insertion lines were tested for each construct.

### Immunocytochemistry

Anti-β-galactosidase immunostaining was performed on embryos containing P[52a-Gal4], P[UAS-Reaper], P[UAS-LacZ], and a P[UAS-Morgue*] insertion to assess CNS midline cell survival. Immunostaining was performed essentially as described in [32]. A primary anti-β-galactosidase Mab (Promega Corp; Madison, WI) was used at a 1∶1000 dilution and a goat biotinlyated anti-mouse secondary antibody (Jackson ImmunoResearch Lab. Inc.; West Grove, PA) was used at a 1∶400 dilution. Detection of secondary antibody localization was performed using a Vectastain HRP ABC detection kit (Vector Labs; Burlingame CA) and samples visualized and photographed using DIC optics on a Nikon Optiphot compound microscope.

### Co-immunoprecipitation of morgue-associated proteins

100–200 P[*da*-Gal4], P[UAS-3xFlag:Morgue] or P[*da*-Gal4], P[UAS- Morgue:3xFlag] adult flies were homogenized in 400 ul lysis buffer (50 mM HEPES, pH 7.5, 150 mM NaCl, 1%Triton X-100, 1 mM phenylmethanesulfonylfluoride (PMSF) and protease inhibitor cocktail (Roche Applied Science; Indianapolis, IN)) and centrifuged at 14,000 rpm at 4°C for fifteen minutes. The supernatant was transferred to a new tube and lysis buffer added to one ml. 30 ul of M2 anti-FLAG resin (Sigma-Aldrich) was added to the supernatant and the sample was rotated for two hours at 4°C. The resin was washed ten times with 500 ul of wash buffer (20 mM Tris-Cl, pH 7.5, 150 mM NaCl, 1 mM PMSF) and then eluted with 3xFLAG peptide (Sigma-Aldrich). The resin was washed with 500 ul of wash buffer five times at room temperature and eluted by directly boiling with 1× SDS protein sample buffer (Thermo Scientific; Rockford, IL) for five minutes.

### Western blots and silver staining

Immunoprecipitated protein samples were mixed with 5x Lane Marker Sample Buffer (Thermo Scientific), boiled for five minutes and electrophoresed on pre-cast SDS/PAGE gels (Thermo Scientific). After separation on SDS/PAGE gels, the samples were transferred to a nitrocellulose membrane (GE Healthcare Life Sciences; Piscataway, NJ). The membranes were blocked in 5% powdered milk in TBST (20 mM Tris-HCl pH 7.5, 150 mM NaCl, 0.5% Tween-20) at room temperature for one hour before being probed with one of the following primary antibodies: anti-FLAG M2 monoclonal antibody (1∶10000 Sigma-Aldrich), anti-ubquitin monoclonal antibody (1∶1000 Santa Cruz Biotechnology; Santa Cruz, CA), anti-ubiquitin polyclonal antibody (1∶750 Dako; Carpenteria, CA), anti-SkpA (1∶750), anti-MBP (1∶750 New England Biolabs; Ipswich MA) at 4°C overnight or at room temperature for 2 hours. This was followed by incubation with one of the following secondary antibodies: HRP (horse radish preoxidase) conjugated goat anti-mouse (1∶10000) (GE Healthcare) or HRP conjugated goat anti-rabbit (1∶10000) (Rockland Immunochemicals Inc.; Gilbertsville, PA). All primary and secondary antibodies were diluted with 1% BSA in TBST. And membranes were washed in TBST for 25 minutes with five separate buffer changes at five minute intervals. Protein bands were visualized using chemiluminescence substrates or a SuperSignal West Dura Chemiluminescence Substrate kit (Thermo Scientific). When using the SuperSignal kit, secondary antibody dilutions were as follows: 1∶2500 for HRP conjugated anti-mouse (GE Healthcare), 1∶2500 for HRP conjugated anti-rabbit (Rockland Immunochemicals Inc.), and 1∶5000 for Mouse TrueBlot® ULTRA: HRP conjugated Anti-Mouse (eBioscience; San Diego, CA). For stripping and re-probing, the membranes were washed in 1% SDS for 1 hour.

For silver staining, after a brief rinse in distilled water, SDS/PAGE gels were incubated at room temperature in fixing solution (50% methanol, 12% acetic acid, 0.05% formaldehyde) for 100 minutes, followed by three 20 minute washes in 50% EtOH. The gel was pre-incubated for 1-minute in 0.2 mg/ml (0.12 M) Na_2_S_2_O_3_ followed by a 20-minute incubation in 4 mg/ml (0.024 M) AgNO_3_ before final incubation in developing solution (60 mg/ml (0.55 M) Na_2_CO_3_, 4 ug/ml (0.026 M) Na_2_S2O_3_, 0.05% formaldehyde). Between each step, the gels were subjected to three 20 second washes in distilled water. When the appropriate level of staining was observed the gels were transferred into 10 mM EDTA, pH 8.0 to stop the staining reaction.

### Mass spectrometry

For mass spectrometry analysis, 200 ul of the co-immunoprecipitation eluates from three independent experiments were concentrated via rotary evaporator to 40 ul, electrophoresed using precast 4–20% gradient SDS polyacrylamide gels (Pierce, Rockford IL), and stained by Imperial Protein Stain (Thermo Scientific). The three most significant and specific bands were excised via razor blades and stored in 50 ul of distilled water. The mass spectrometry analysis was conducted by the Center for Advanced Proteomics Research (http://njms.umdnj.edu/proweb/; New Jersey Medical School; Newark, NJ).

### Expression and purification of MBP-morgue fusion proteins

Coding sequences for full length Morgue (Morgue-FL), N-terminus Morgue (Morgue-N: residues 1–301), or C-terminus Morgue (Morgue-C: residues 302–491) were amplified using a full length Morgue cDNA clone as template. A TEV (Tobacco Etch Virus) protease recognition site was incorporated in the 5′ primer so that Morgue sequence is flanked by 1×TEV sequence at the 5′ end. The stop codon was removed from the 3′ primers of the coding sequences to create a read-through. The TEV-Morgue PCR product was subcloned into the pET21d vector (EMD Chemicals; Philadelphia, PA) using EcoRI and XhoI sites to fuse the 6xHis tag into the open reading frame. The maltose-binding protein (MBP) coding sequence was amplified by PCR, and a second TEV was incorporated into the 3′ primer. This PCR product was then inserted into the pET21d-TEV-Morgue construct, using NcoI and EcoRI sites. The fidelity of the resulting constructs was verified by DNA sequencing (Davis Sequencing).

The constructs containing MBP-tagged full length or truncated Morgues were transformed into *E. coli* Rosetta strain (EMD chemicals). A single colony was inoculated into 5 ml LB culture and grown at 37°C overnight. The overnight culture was subsequently inoculated into 250 ml LB media (with 50 ug/ml Amp and 0.2% glucose) and grown at 37°C for 4 hours. IPTG was added into the culture at a final concentration of 0.5 mM and induction was continued at 30°C for 4–6 hours. The cells were spun down at 8,000 rpm for 15 minutes and resuspended in 15–20 mls of column buffer (20 mM Tris-HCl, pH 7.5, 200 mM NaCl, 0.2% Triton X-100, 1 mM PMSF, and 1× protease inhibitor cocktail (Roche Applied Science)) and sonicated on ice 3–4 times at an interval of 40 seconds. Cell lysate was centrifuged at 13000 rpm for 15 minutes and supernatant was collected and diluted to 50 ml with column buffer. The diluted cell lysate was saved at −20°C for ubiquitin binding assay.

Cell lysate was then loaded onto an amylose resin column (New England Biolabs), washed with 12 volumes of wash buffer (20 mM Tris-HCl, 500 mM NaCl, 0.2%Triton X-100, 1 mM PMSF), and eluted with 3 volumes of elution buffer (10 mM maltose in 20 mM Tris-HCl, pH 7.5, 200 mM NaCl). The eluate was collected and diluted to 25 ml with binding buffer (50 mM Tris-HCl, pH 8.0, 300 mM NaCl, 5 mM imidazole) and loaded on a Ni-NTA agarose column (EMD Chemicals). The loaded column was washed with 5 volumes of wash buffer (50 mM Tris-HCl, pH 8.0, 300 mM NaCl, 20 mM imidazole), and eluted with elution buffer (50 mM Tris-HCl, pH 8.0, 300 mM NaCl, 500 mM imidazole). All steps were performed at 4°C.

### Ubiquitin binding assay

10 ml of cultured MBP-Morgue-expressing *E. coli* cell lysate (isolated as described in previous section) were incubated with 500 ul of amylose resin (New England Biolabs) at 4°C for 1 hour. The resin was washed 5 times with wash buffer (20 mM HEPES, pH 7.5, 500 mM NaCl, 1 mM PMSF) and 2% of the resin was boiled with 50 ul of 1× Lane Marker Sample Buffer (Pierce) for 5 min; this aliquot was used to examine the purified protein. Resin containing approximately 2 ug of immobilized MBP-Morgue proteins was incubated with 3 ug of K48 polyubiquitin (n = 2–7) (Enzo Life Science, Farmingdale, NY) in 200 ul of binding buffer (20 mM HEPES, pH 7.5, 150 mM NaCl, 0.2% EDTA, 1 mM PMSF, 1× protease inhibitor cocktail (Roche Applied Science)) at 4°C for 1 hour. Binding buffer was then added to a total volume to 500 ul and DSP crosslinker was added to each reaction to a final concentration of 500 uM. This reaction was incubated at room temperature for 30 min. 1 M Tris-HCl, pH 7.5 was added to each reaction at a final concentration at 30 mM to stop the reaction and the samples were rotated at room temperature for 15 min. Resin was washed 5 times with wash buffer (20 mM Tris-HCl, pH 7.5, 50 mM NaCl, 1 mM PMSF) and protein was eluted by boiling for 5 minutes in 1× Lane Marker Sample Buffer (Pierce) containing 5% β-mercaptoethanol.

## Results

### Morgue over-expression induces lethality *in vivo*

Targeted expression of Morgue via the P[GMR-Gal4] driver enhances the cell death induced by Grim-Reaper proteins [26]. To analyze the effects of more widespread Morgue over-expression we expressed Morgue using P[*da*-Gal4] and P[UAS-Morgue] fly strains. The *da* (*daughterless*) gene is expressed in most or all tissues [33], [34]. Both P[*da*-Gal4] and P[UAS-Morgue] strains are fully viable and recombinant chromosomes containing both insertions were generated and maintained as fertile, balanced stocks. Strikingly, homozygous P[*da*-Gal4], P[UAS-Morgue] flies were found to exhibit a completely lethal phenotype at 25°C (Table 1). Lethality was typically associated with late 3^rd^ instar larvae and pupae. To confirm that this lethality results specifically from Morgue expression, similar crosses were performed to generate P[*da*-Gal4], P[UAS-GFP] flies. Homozygous P[*da*-Gal4],P[UAS-GFP] flies did not exhibit reduced viability, strongly suggesting that ectopic Morgue expression specifically induces lethality. In addition, we also examined flies carrying two copies of P[*da*-Gal4] and one copy of P[UAS-Morgue] or vice versa. The use of two different independent P[UAS-Morgue] insertion lines resulted in complete or nearly complete lethality of these semi-homozygotes (Table 1). This variation likely reflects dosage-dependence and position effects of the sites of the P[UAS-Morgue] insertion strain utilized (Note: only a single P[*da*-Gal4] line was utilized) or genetic background differences between the recombinant P[*da*-Gal4], P[UAS-Morgue] chromosomes. It is likely that flies that express higher levels of Morgue exhibit increased lethality. While it is not yet clear what the relevant Morgue activities or specific sites of expression are for this lethality, this phenotype provides a useful assay for Morgue function.

**Table 1:** Overexpression of Morgue results in lethality.

parents		offspring			
female	male	+	balancer	+/balancer ratio	ratio if fully viable
da-Gal4/da-Gal4	da-Gal4/TM3	176	162	1.09	1.00
da-Gal4/TM3	da-Gal4/TM3	310	529	0.59	0.50
da-Gal4, UAS-GFP/TM3	da-Gal4, UAS-GFP/TM3	530	1191	0.45	0.50
da-Gal4, UAS-morgue1/TM3	da-Gal4, UAS-morgue1/TM3	0	1278	0.00	0.50
da-Gal4, UAS-morgue2/TM3	da-Gal4, UAS-morgue2/TM3	0	975	0.00	0.50
da-Gal4/da-Gal4	da-Gal4,UAS-morgue1/TM3	50	238	0.21	1.00
da-Gal4/da-Gal4	da-Gal4,UAS-morgue2/TM3	0	183	0.000	1.00
da-Gal4, UAS-morgue1/TM6B	UAS-morgue1/UAS-morgue1	1	83	0.012	1.00
da-Gal4, UAS-morgue1/TM3	UAS-morgue1/UAS-morgue1	1	180	0.006	1.00
da-Gal4, UAS-morgue2/TM3	UAS-morgue1/UAS-morgue1	32	238	0.13	1.00

Effects of widespread Morgue expression on fly viability. Counts of heterozygote (balancer) and homozygote non-balancer (+) progeny derived from genetic crosses where P[*da*-Gal4] was used to drive expression of P[UAS-GFP] or P[UAS-Morgue]. Homozygous P[*da*-Gal4] or P[*da*-Gal4],P[UAS-GFP] flies are viable while P[*da*-Gal4],P[UAS-Morgue] homozygotes are completely lethal. Flies containing two copies of P[*da*-Gal4] and one copy of P[UAS-Morgue] or vice versa exhibit either complete or significant lethality. UAS-morgue1 and UAS-morgue2 represent independent insertions of P[UAS-Morgue].

### Morgue-induced lethality requires functions of all three conserved domains

Using the Morgue-induced lethality phenotype as a functional assay, we dissected the importance of each domain/motif of the Morgue protein. Individual or multiple domains/motifs were deleted in Morgue (Figure 1A) and the viability of homozygous P[*da*-Gal4], P[UAS-MorgueΔ] (Δ = deletion) flies were examined (Table 1). Specific deletion of the zinc finger (MorgueΔZF), F box (MorgueΔFB), or UEV domain (MorgueΔUEV) resulted in reduced levels of Morgue-induced lethality (Table 2) with independent transformants exhibiting a range of effects from full lethality (MorgueΔFB(2)) to approximately 80% viability (MorgueΔUEV(2)). Expression of Morgue mutants lacking the F box and UEV domain (MorgueΔFB-UEV) or the zinc finger and F box (MorgueΔZF-FB) also exhibited reduced lethality compared to native, full length Morgue (Table 2). These results indicate that all three of the conserved Morgue domains, the zinc finger, F box, and UEV domain contribute to the lethal phenotype associated with Morgue over-expression phenotype, as deleting any (or combinations) of these domains attenuates the ability of Morgue to induce lethality. However, none of these individual domains is fully responsible for the Morgue-induced lethal phenotype as their loss does not fully eliminate lethality and restore complete viability.

**Table 2:** Overexpresion of Morgue deletion (MorgueΔ) mutants exhibit variable viability.

UAS-linked gene	+	balancer	+/balancer ratio	ratio if fully viable
GFP	530	1191	44.5%	50%
Morgue (1)	0	1278	0%	50%
Morgue (2)	0	975	0%	50%
MorgueΔZF (1)	568	1866	30.4%	50%
MorgueΔZF (2)	385	1614	23.9%	50%
MorgueΔFB (1)	43	1587	2.7%	50%
MorgueΔFB (2)	0	471	0%	25%
MorgueΔUEV (1)	408	1591	25.6%	50%
MorgueΔUEV (2)	593	1509	39.3%	50%
MorgueΔ (FB+UEV)	651	1750	37.2%	50%
MorgueΔ (ZF+FB) (1)	82	484	16.9%	25%
MorgueΔ (ZF+FB) (2)	55	296	18.6%	25%

Effects of widespread expression of Morgue deletion mutants on fly viability. Counts of homozygous non-balancer (+) and heterozygote balancer progeny derived from genetic crosses where P[*da*-Gal4] was used to drive expression of P[UAS-GFP] or various P[UAS-MorgueΔ] strains. Homozygote (non-balancer) and heterozygote (with either a TM3 or both a CyO and a TM3 balancer) progeny derived from: P[*da*-Gal4], P[UAS-MorgueΔ]/TM3, P[*da*-Gal4], P[UAS-GFP]/TM3, or P[UAS-MorgueΔ]/CyO, P[*da*-Gal4]/TM3 parent flies were counted. Expression of MorgueΔZF, MorgueΔFB, MorgueΔUEV, and MorgueΔZF-FB resulted in reduced Morgue-induced lethality. In contrast, removal of the F box alone retained near complete Morgue-induced lethality. Note that if the homozygotes are fully viable, the expected percentage of viable flies is either 50% or 25% depending on whether one or two balancer chromosomes are present.

### The zinc finger and F box are not essential for morgue’s ability to enhance R/Grim-induced eye cell death

The Morgue deletion proteins were also examined for their abilities to enhance the eye cell death induced by expression of a chimeric R/Grim protein. P[GMR-Gal4], P[UAS-R/Grim] flies exhibit a moderate eye cell death phenotype that includes reduction in size and pigmentation of the adult compound eye. The presence of P[UAS-Morgue] enhances the level of eye cell death observed in P[GMR-Gal4], P[UAS-R/Grim] flies (Figure 1B; compare GFP to Morgue) in a manner similar to that observed for the *morgue* EP2367 chromosome (Wing et al. 2002). Deletion of the zinc finger or F box did not significantly disrupt Morgue’s ability to enhance cell death as expression of MorgueΔZF, MorgueΔFB, and MorgueΔUEV all exhibited essentially the same R/Grim-induced eye phenotypes as native Morgue (Figure 1B). Thus, the zinc finger, F-box, or UEV domain are not individually essential for Morgue’s ability to enhance eye cell death, as removal of each single domain does not dramatically alter the enhanced eye cell death phenotype.

### The conserved Gly421 in the UEV domain is not critical for morgue over-expression phenotypes

**Figure 2:**
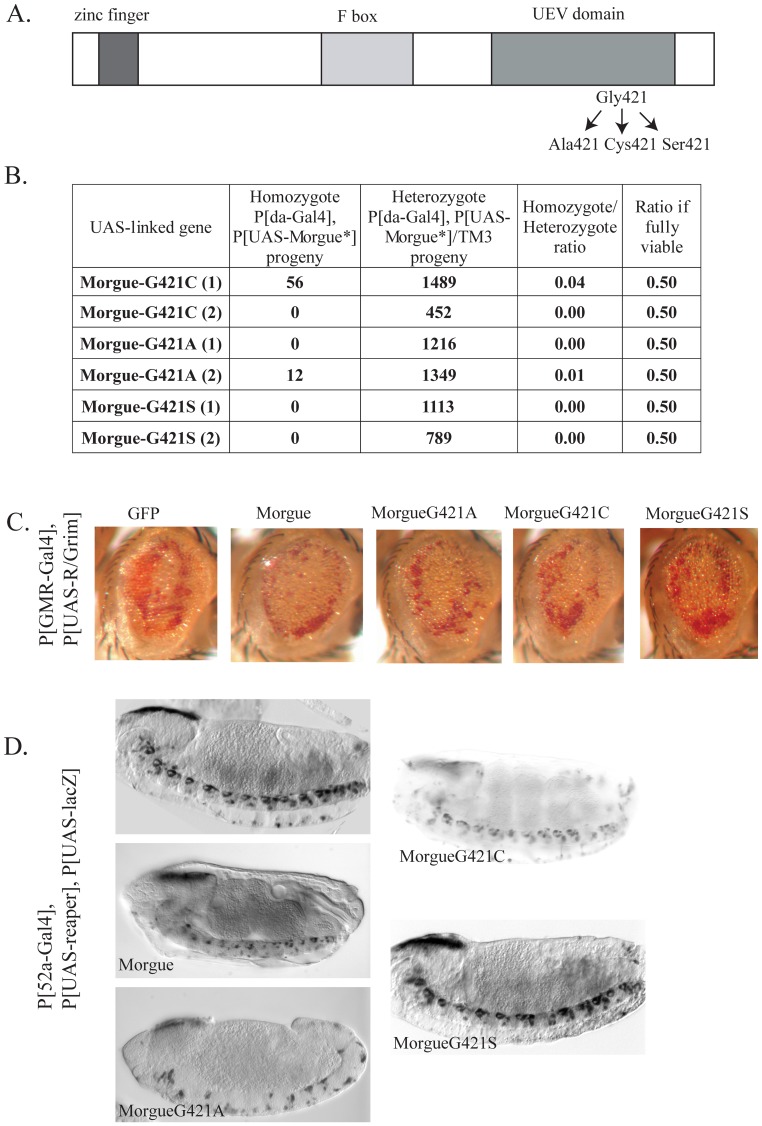
Substitutions of the Morgue Gly421 residue do not affect Morgue-induced lethality or cell death. **A.** Schematic representation of Morgue point mutant proteins with Alanine, Cysteine, or Serine substitutions of the active site Glycine (residue 421). **B.** The progeny derived from P[*da*-Gal4], P[UAS-MorgueG421X]/TM3 parent flies were examined and the percentage of homozygotes/heterozygotes was determined. As for native Morgue, expression of each Morgue point mutant essentially resulted in completely lethality. Note that the expected percentage of homozygotes/heterozygotes is 50% if the homozygotes are fully viable. **C.** Enhancement of eye cell death in P[GMR-Gal4], P[UAS-R/Grim] flies by Morgue point mutants. Compared to GFP co-expression of native Morgue with R/Grim results in enhanced levels of eye cell death (evidenced by additional loss of pigment cells). Similar enhancement is observed for co-expression of the MorgueG421A, MorgueG421C, and MorgueG421S point mutants. **D.** Enhancement of Reaper-induced CNS midline cell death by Morgue Gly421 mutant proteins. The P[52a-Gal4] line was used to drive expression of P[UAS-LacZ] and P[UAS-Reaper] in embryonic CNS midline cells. Expression of Reaper alone does not induce significant amounts of cell death. Co-expression of native Morgue induces increased levels of CNS midline cell death. This enhanced death is somewhat enhanced by Morgue421A but is not significantly altered by either the MorgueG421C or MorgueG421S point mutant proteins. All views are sagittal with anterior to left. Stage 12 (P[UAS-Reaper] and PUAS-Morgue421A]), stage 16 (P[UAS-Morgue and P[UAS-MorgueG421C]), and stage15 (P[UAS-Reaper] and P[UAS-MorgueG421S]) embryos are shown.

The invariance of the Gly421 in the catalytic site of the Morgue UEV domains suggests that this Glycine residue may have specific and crucial functions. To test this possibility we generated P[UAS-MorgueG421x] lines (Figure 2A) with a mis-sense point mutation of Gly421 to a Cysteine (G421C: the active catalytic residue in bona fide E2 conjugases), an Alanine (G421A: a small hydrophobic, non-polar amino acid chemically similar to Glycine), or a Serine (G421S: a polar residue present in other UEVs). The Morgue point mutant proteins were examined for their ability to induce lethality when expressed by P[*da*-Gal4], and their ability to enhance R/Grim eye cell death in P[GMR-Gal4], P[UAS-R/Grim] flies. All three mutants exhibit complete or nearly complete lethality when expressed as homozygotes by P[*da*-Gal4] (Figure 2B). Thus, the invariant Gly421 residue is not essential for Morgue-induced lethality.

Each of the Gly421x mutant Morgue proteins also exhibited a similar ability as native Morgue to enhance eye cell death in P[GMR-Gal4], P[UAS-R/Grim] flies (Figure 2C; data not shown for P[UAS-MorgueG421S]). The Morgue point mutants were also examined for their ability to enhance *reaper*-induced CNS midline cell death using the P[52a-Gal4] line. Co-expression of *reaper* and *morgue* (via EP 2367) in the embryonic midline via P[52A-Gal4] results in increased midline cell death compared to expression of *reaper* alone (Wing et al., 2002). Wild-type Morgue as well as Morgue Gly421x mutants were co-expressed with Reaper in P[52A-Gal4], P[UAS-LacZ] flies and anti-ß-galactosidase immunostaining was used to label the midline cells in embryos. The results revealed that the Morgue Gly421x mutants exhibit a similar cell death-enhancing phenotype as native Morgue (Figure 2D; data now shown for P[UAS-MorgueG421A]). These data indicate that the conserved Gly421 residue is not specifically essential for enhancement of Grim-Reaper mediated cell death.

### Targeted expression of 3xFLAG tagged morgue fusion proteins exhibit similar phenotypes as native morgue protein

**Figure 3:**
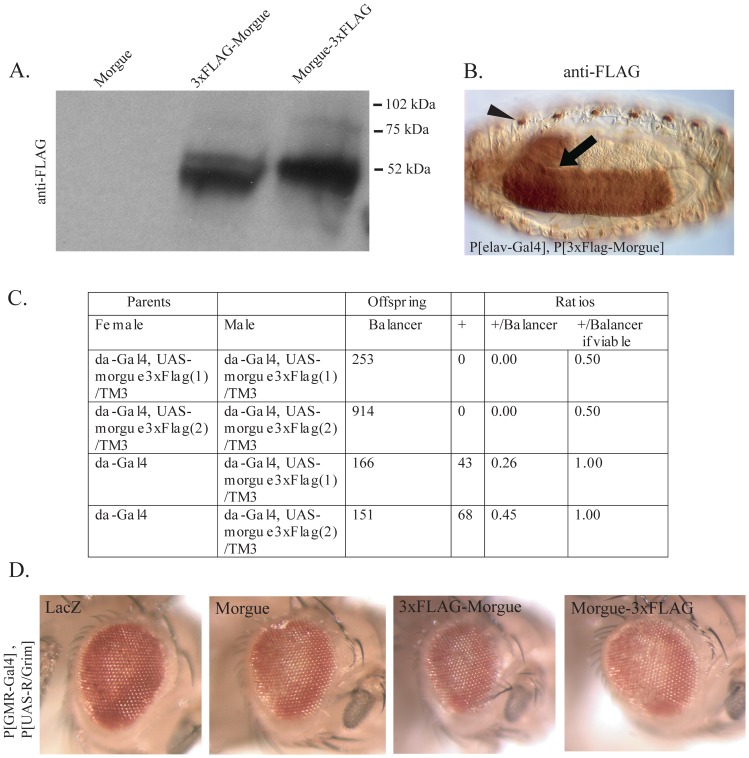
3xFLAG-Morgue is expressed and functional in fly tissues. **A.** The P[Elav-Gal4] or P[*da*-Gal4] lines were used to express Morgue-3xFLAG (P[UAS-Morgue:3xFlag]) or 3xFLAG-Morgue (P[UAS-3xFlag:Morgue]) in different independent insertion lines. Cell free lysate were analyzed by SDS/PAGE and anti-FLAG Western blots. No anti-FLAG immunoreactive band was observed in a negative control using P[*elav*-Gal4] and P[UAS-*morgue*]. **B.** Anti-Flag immunostaining of stage 16 embryos carrying a copy of P[*elav*-Gal4] and P[UAS-3XFlag:Morgue]. 3XFLAG-Morgue protein is highly expressed in the embryonic central (arrow) and peripheral (arrowhead) nervous system. **C.** Viability of flies expressing 3xFLAG-Morgue via P[*da*-Gal4]. Progeny from crosses between flies carrying different combinations of P[*da*-Gal4] and P[UAS-3xFlag:Morgue] or P[UAS-Morgue:3xFlag] were analyzed. Flies homozygous for P[*da*-Gal4] and P[UAS-Morgue:3xFlag] are completely lethal. Flies with reduced copies of P[*da*-Gal4] or P[UAS-Morgue:3xFlag] exhibit variable effects ranging from nearly full lethality to nearly full viability. **D.** Expression of Morgue-3xFLAG and 3xFLAG-Morgue proteins induce a similar cell death enhancement phenotype as that observed for wild-type Morgue when co-expressed with R/Grim. LacZ-P[GMR-Gal4],P[UAS-R/Grim] and P[UAS-LacZ]: Morgue-P[GMR-Gal4],P[UAS-R/Grim] and P[UAS-Morgue]: 3xFLAG-Morgue-P[GMR-Gal4],P[UAS-R/Grim] and P[UAS-3xFlag:Morgue]: and Morgue3xFLAG-P[GMR-Gal4],P[UAS-R/Grim] and P[UAS-Morgue:3xFlag]. Both Morgue-3x-FLAG and 3x-FLAG-Morgue proteins exhibit a similar ability to enhance R/Grim induced cell death in the eye as wild-type Morgue. All photos were taken of 1–3 day old flies.

Over-expression of Morgue alters organismal viability and enhances cell death. Because the Morgue protein contains several putative protein interaction domains, these phenotypes likely involve interactions between Morgue and other factors. To identify these factors we performed *in vivo* co-immunoprecipitation experiments to identify Morgue-associated proteins in adult flies. P[UAS-3xFlag:Morgue] and P[UAS-Morgue:3xFlag] were generated that express full length wild-type Morgue proteins containing a 3xFLAG epitope tag at either the NH_2_- or COOH-terminal end. Two approaches were used to confirm that these lines both express active fusion proteins in a Gal4-dependent fashion. The first approach was to analyze the expression of FLAG:Morgue and Morgue:FLAG proteins. P[UAS-Morgue], P[UAS-3xFlag:Morgue], and P[UAS-Morgue:3xFlag] were crossed to P[*da*-Gal4] and P[*elav*-Gal4] strains. Protein extracts from the adult progeny were analyzed via anti-FLAG Western blots. Prominent bands were observed at the predicted molecular weight of ∼60 kDa in FLAG:Morgue- and Morgue:FLAG-expressing samples (Figure 3A). Anti-FLAG immunohistochemistry was also performed on embryos derived from crosses between P[*elav*-Gal4] and P[UAS-3xFlag:Morgue] or P[UAS-Morgue:3xFlag]. Strong immunostaining was observed for both 3xFLAG:Morgue and Morgue:3xFlag throughout the central and peripheral nervous system (Figure 3B and data not shown). These results confirm that the tagged Morgue proteins are appropriately expressed.

The second approach to analyze the Morgue:FLAG proteins was functional. Over-expression of P[Morgue:3xFlag] was examined for lethality when expressed by P[*da*-Gal4]. Homozygous P[*da*-Gal4], P[UAS-Morgue:3xFlag] flies exhibited the same complete lethality as homozygous P[*da*-Gal4], P[UAS-Morgue] flies (Figure 3C). Both P[UAS-3xFlag:Morgue] and P[UAS-Morgue:3xFlag] were also analyzed for their ability to enhance the eye cell death phenotype of P[GMR-Gal4]/P[UAS-R/Grim] flies. Targeted expression of each fusion protein was shown to exhibit similar eye cell death enhancement as native Morgue (Figure 3D). In addition, similar to native Morgue, both P[3xFlag:*morgue*] and P[Morgue:3xFlag] enhanced P[UAS-Reaper]-induced cell death in the embryonic CNS midline (data not shown). Taken together, the data indicate that expression of each Morgue fusion protein can be targeted specifically and that Morgue functions are not impaired by the 3xFLAG tag. The Morgue:FLAG proteins can be expressed at high levels and are functional. They thus represent useful reagents for identifying Morgue-associated proteins.

### Morgue associates with SkpA and K48 linked polyubiquitin *in vivo*

**Figure 4:**
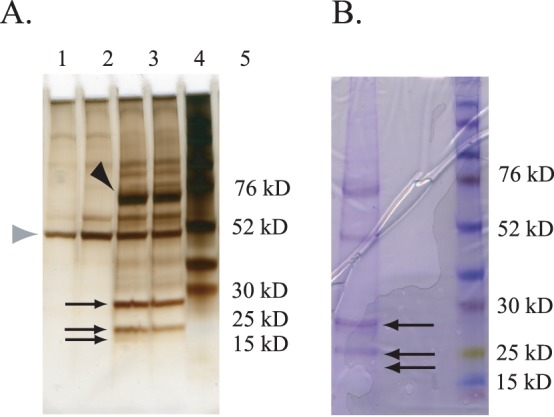
Purification of three Morgue-associated proteins. **A.** Silver staining of an analytical SDS polyacrylamide gel separating proteins from whole fly extracts that associate with Morgue. Protein bands corresponding to Morgue-3xFLAG (∼60 kD) (black arrowhead) as well as the anti-FLAG Ig heavy chain (∼50 kD) are indicated (gray arrowhead). Lanes 1–4 correspond to material purified via the anti-FLAG resin from the following flies: Lane 1: P[*da*-Gal4]; Lane 2: P[UAS-Morgue3xFlag]; Lane 3: P[*da*-Gal4], P[UAS-3xFlag:Morgue]; Lane 4: P[*da*-Gal4], P[UAS-Morgue3xFlag]; Lane 5: Protein molecular weight markers. The three major bands corresponding to Morgue-associated proteins purified are indicated (arrows) and correspond to polypeptides migrating between 28 kD and 20 kD. **B.** Coomassie Brilliant Blue staining of a preparatory SDS polyacrylamide gel separating Morgue-associated proteins from extracts of P[*da*-Gal4], P[UAS-Morgue:3xFlag] adult flies purified via anti-FLAG resin. Three major bands (arrows) were excised from the gel and the corresponding polypeptides analyzed via mass spectrometry. Lane on right corresponds to protein molecular weight markers.

**Figure 5:**
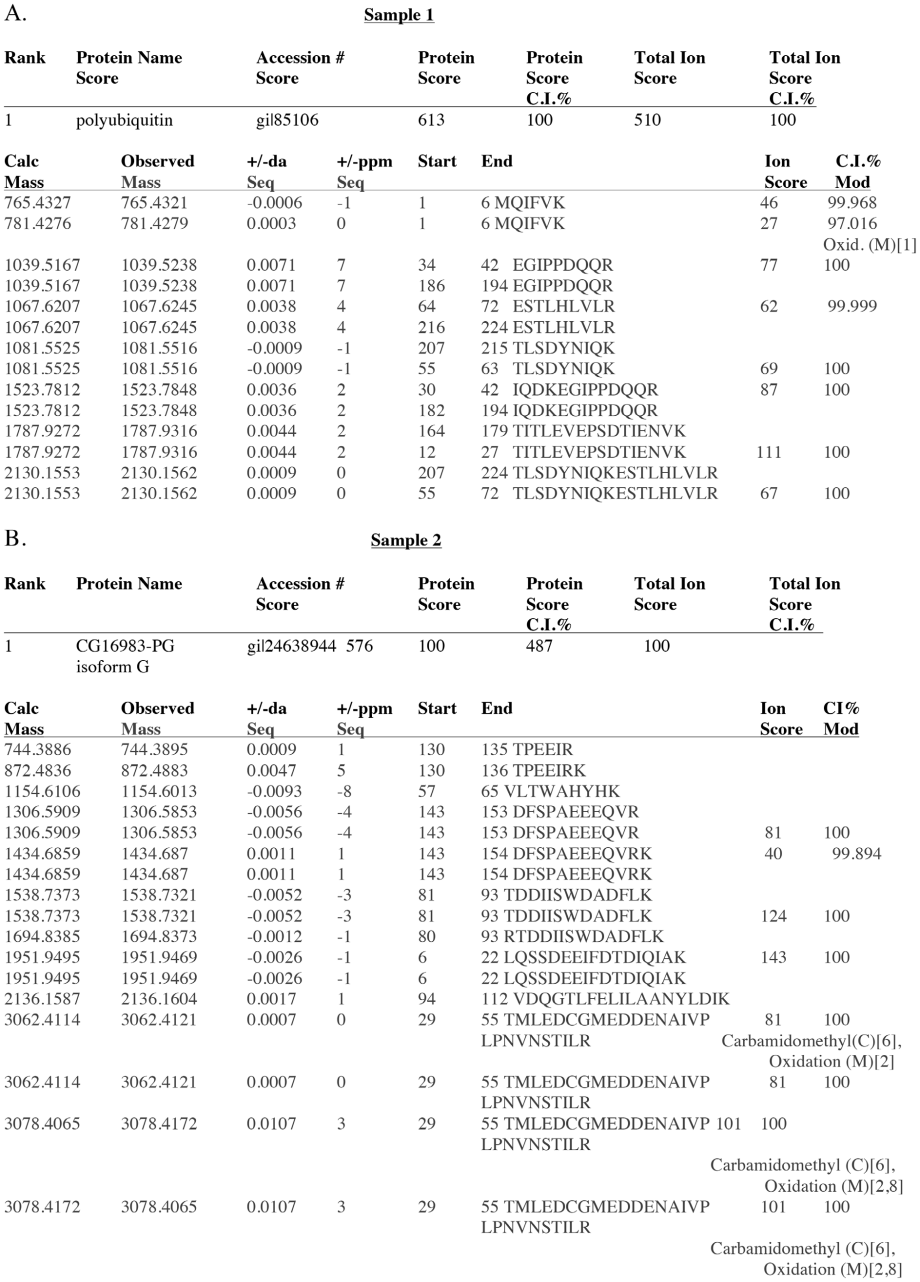
Identification of Morgue-associated proteins isolated using P[UAS-Morgue3xFLAG] in adult flies. **A.** Amino acid sequence analysis of tryptic peptides derived from protein sample 1 indicates Morgue associates with a ubiquitin multimer. Molecular mass and position of peptide sequences are indicated. Note repeated peptide sequences present at distinct positions along sample 1 protein. **B.** Amino acid sequence analysis of tryptic peptides derived from protein sample 2 indicates Morgue associates with SkpA. Molecular mass and position of peptide sequences are indicated.

**Figure 6:**
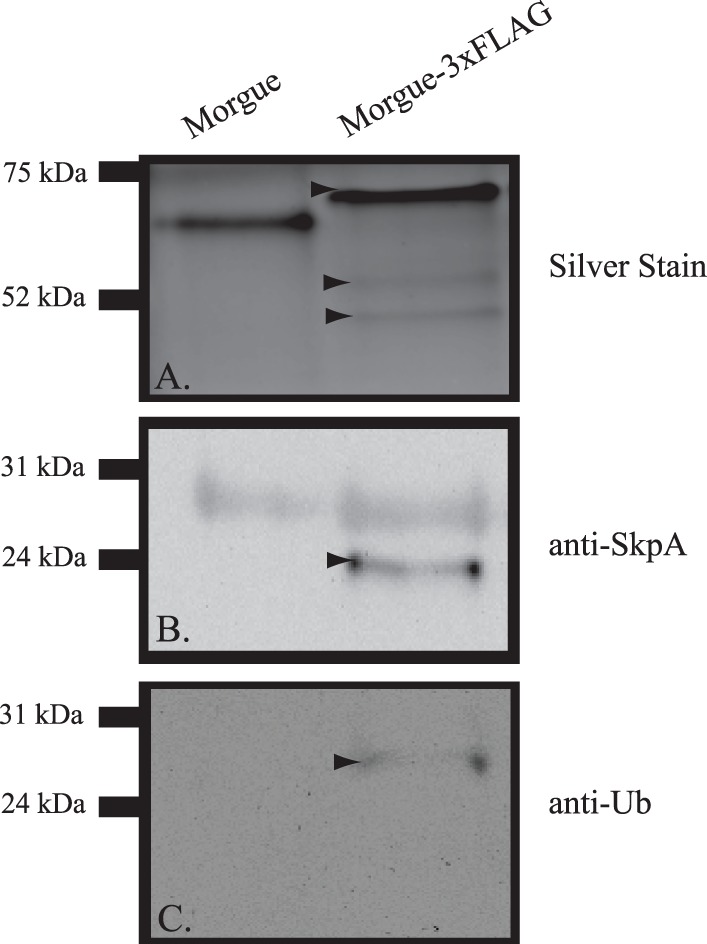
Western blot analysis of Morgue-associated proteins confirms Morgue association with polyubiquitin and SkpA. **A.** Silver stained SDS gel of proteins from P[*da*-Gal4], P[UAS-Morgue] or P[*da*-Gal4],P[UAS-Morgue-3xFLAG] adult flies. Note that apparent molecular weight of Morgue-3xFLAG (arrowhead) is greater than native Morgue. Note smaller proteins (lower arrowheads) that are present in Morgue-3xFLAG expressing flies. **B.** Anti-SkpA Western blot of proteins purified from P[*da*-Gal4], P[UAS-Morgue] or P[*da*-Gal4], P[UAS-Morgue:3xFlag] flies via anti-FLAG chromatography. Note prominent SkpA band (arrowhead) present in proteins purified from P[*da-*Gal4], P[UAS-Morgue:3xFlag]. A shared background band of approximately 30 kDa is present in both samples. **C.** Anti-ubiquitin Western blot of proteins purified from P[*da*-Gal4], P[UAS-Morgue] or P[*da*-Gal4], P[UAS-Morgue:3xFlag] flies via anti-FLAG chromatography. Note ubiquitin band (arrowhead) present in proteins purified from P[*da*-Gal4], P[UAS-Morgue:3xFlag].

To isolate proteins that associate with Morgue *in vivo*, P[UAS-3xFlag:Morgue] and P[UAS-Morgue:3xFlag] flies were crossed to P[*da*-gal4] flies. Protein extracts were isolated from progeny flies and subjected to anti-FLAG affinity chromatography. The eluate was analyzed via SDS polyacrylamide gel electrophoresis and silver staining. Three major bands between 20 kDa to 30 kDa co-precipitated specifically with both 3xFLAG:Morgue and Morgue:3xFLAG but not native Morgue lysates (Figure 4A). To isolate these proteins, the eluates were separated on preparative SDS gels and lightly stained with Coommassie Blue (Figure 4B). The three co-precipitating bands were purified and subjected to digestion with trypsin and analyzed via MALDI-TOF/TOF mass spectrometry (Figure S1). Analysis of the trypsin digestion products indicated that the three bands corresponded to two distinct polypeptides. Peptide sequence analyses revealed that the smaller two bands (∼20 kDa) both correspond to SkpA while the larger band (∼28k Da) corresponds to a polyubiquitin moiety (Figure 5A,B). Morgue association with SkpA and polyubiquitin was confirmed via anti-SkpA and anti-ubiquitin Western blots of Morgue co-immunoprecipitation extracts (Figure 6A–C). Association between Morgue and SkpA was previously detected via *in vitro* GST-pulldown [26] and yeast 2-hybrid [35] assays. In contrast, association between Morgue and ubiquitin has not been previously reported. The apparent molecular weight of the ubiquitin moiety suggests that Morgue associates with a ubiquitin multimer. One possibility is that this ubiquitin multimer corresponds to a free linear polyubiquitin chain encoded by a polyubiquitin gene. However, the two *Drosophila* polyubiquitin genes, Ubi-p5E (CG32744) and Ubi-p63E (CG11624), encode seven and eighteen ubiquitin repeats respectively [36], [37]. These polyubiquitin proteins are significantly larger than the Morgue-associating ubiquitin multimer. Furthermore, linear polyubiquitin is typically cleaved into ubiquitin monomers co-translationally, before the entire polyubquitin peptide is translated [38]. Thus it is unlikely that the Morgue-associated ubiquitin oligomer derives from genetically encoded polyubiquitin. It is more likely that Morgue associates with a ubiquitin chain generated post-translationally by the actions of a ubiquitin E1 activator, E2 conjugase, and E3 ligase.

There are 7 distinct Lysine residues (K6, K11, K27, K29, K33, K48, and K63) in the ubiquitin molecule. For each of these, the lysine ?-NH_2_ group can form an isopeptide linkage to the COOH-terminal Glycine residue of a separate ubiquitin moiety. In addition, the NH_2_-terminal Methionine may also be used as a ubiquitin addition site. Thus, there are eight potentially distinct stoichiometric structures for di-ubiquitin, seven branched and one linear [39], [40]. Interestingly, different linkages of polyubiquitin chains mediate distinct biological functions 41, [42] such as targeting proteins to the proteasome (K48 linkage) or DNA repair (K63 linkage). Significantly, tryptic digestions of each linkage-specific polyubiquitin produces a unique, diagnostic peptide [43]. Thus, the organization of a poyubiquitin moiety can be determined by the presence of a characteristic molecular weight peak in mass spectrometry analyses of trypsin digests (Figure S1). In this study, the K48-specific molecular weight peak of 1460.7855 was detected (Figure S1) in the mass spectrometry analysis of tryptic digests. This indicates that the Morgue-associated polyubiquitin is K48-linked. No other diagnostic tryptic peptide was detected; Morgue does not appear to associate with any other polyubiquitin stoichiometries. This result strongly suggests that Morgue regulates ubiquitination of target proteins and targets them for digestion via the 26S proteasome.

### Multiple regions of morgue are required to associate with polyubiquitin *in vitro*

**Figure 7:**
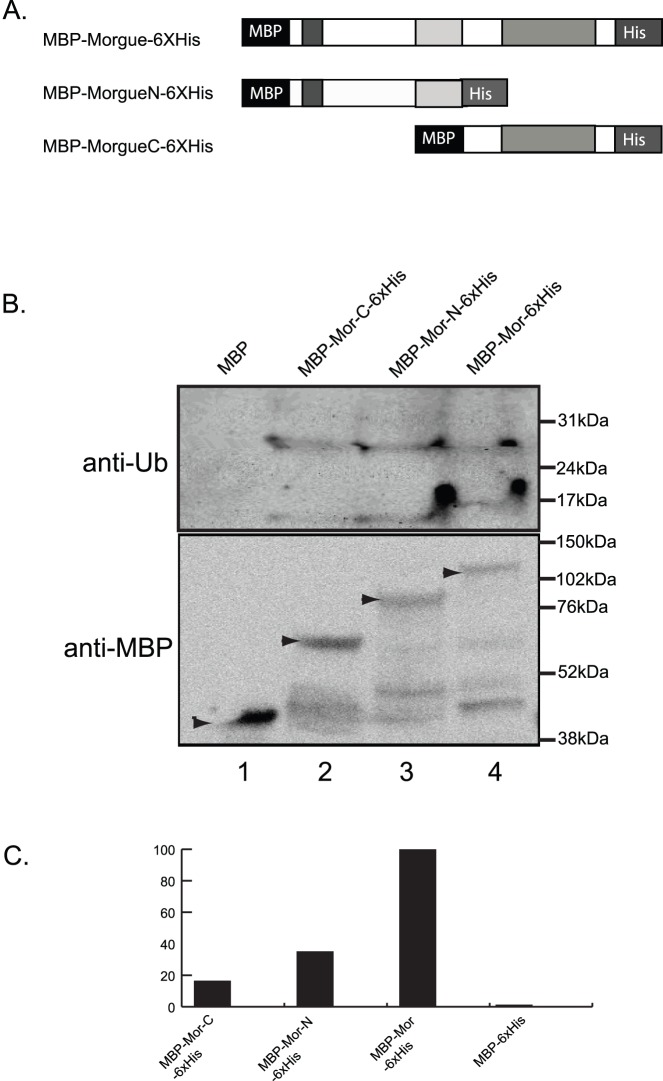
Both the NH_2_ and COOH regions of Morgue protein exhibit association with polyubiquitin. **A.** Schematic representation of MBP (Maltose binding protein) and 6xHis-tagged full length Morgue (MBP:Morgue:6xHis) as well as either the Morgue NH2 (MBP-MorgueN:6xHis) or COOH (MBP:MorgueC:6xHis) region. Note that MorgueN contains the zinc finger and F box while MorgueC contains the UEV domain. **B.** Western blot analysis of *in vitro* immunoprecipitation assays using ubiquitin (anti-Ub) or MBP (anti-MBP) antisera and native or tagged Morgue proteins. Note presence of ubiquitin immunoreactive bands at approximate sizes of ubiquitin dimers and tetramers that associate with tagged Morgues but not native Morgue protein. Also, MBP immunoreactive bands are observed for each tagged Morgue but not native Morgue. The distinct sizes of these bands correspond to distinct sizes of tagged Morgue proteins. **C.** Semi-quantitative analysis of Morgue association with ubiquitin. The tagged MorgueN protein exhibits slightly stronger association to polyubiquitin than the tagged MorgueC protein. Neither exhibits the full level of association seen for full length Morgue. Minimal association is observed between MBP and polyubiqutin.

The basis for association between Morgue and polyubiquitin was further analyzed via *in vitro* assays to determine which domain(s)/motif(s) of Morgue mediate this interaction. To address this question, full length Morgue, as well as Morgue-NH_2_ (zinc finger+F-box) and Morgue-COOH (UEV domain) polypeptides were fused to the maltose binding protein (MBP) at the NH_2_-terminus and a 6xHis tag at the COOH-terminus (Figure 7A). These proteins were expressed in *E. coli* and purified via amylose resin affinity chromatography. The mutant Morgue proteins were each incubated with K48-linked polyubiquitin chains (n = 2–7) in the presence of the protein crosslinker DSP (dithiobis [succinimidyl propionate]) which cross-links amine groups located within a distance of 9–12Å [44]. At a DSP concentration of 500 uM, polyubiquitin was co-precipitated by MBP-Morgue but not by the MBP control. Surprisingly, both the tagged Morgue-NH_2_ and Morgue-COOH proteins also exhibited association with polyubiquitin (Figure 7B) although as not extensively as full length Morgue. This result suggests that multiple domains of Morgue contribute to polyubiquitin association. The NH_2_-terminal region of Morgue contains both the zinc finger and the F-box domain, and from this experiment it was not possible to distinguish which one, if either, of these regions may bind polyubiquitin. However, since zinc finger motifs correspond to one group of ubiquitin binding domains [20]-[22], it is likely that highly conserved Morgue zinc finger is involved in the polyubiquitin binding. The UEV domain also constitutes a defined ubiquitin-binding domain. Analysis of relative binding strengths were performed by analyzing the Western blot signal intensities, and the results suggest stronger binding of polyubiquitin by the Morgue NH_2_-terminus (zinc finger and F-box) than the COOH-terminus (UEV domain) (Figure 7C). In addition, this binding assay indicated that Morgue binds both dimeric and trimeric K48-linked polyubiquitin, indicating that Morgue may associate with distinct sized polyubiquitin chains. Overall, Morgue appears to constitute a novel ubiquitin-binding protein and may contain multiple ubiquitin-binding domains (UBDs).

## Discussion

### In vivo studies reveal dosage- and domain-dependence of morgue induced lethality and cell death enhancement

Ubiquitous over-expression of Morgue protein using P[*da*-gal4] and distinct P[UAS-Morgue] insertions resulted in complete lethality of homozygous animals. In contrast, homozygous P[*da*-gal4], P[UAS-GFP] flies were fully viable. While the precise basis of this Morgue-dependent lethality is not clear and may involve novel Morgue actions, given Morgue’s functions in cell differentiation and survival, it is likely that specific developmental processes are disrupted. This lethality contrasts with the viable phenotype of *morgue* loss-of-function mutants [27] and suggests that proper regulation of Morgue expression is important for animal development. The Morgue over-expression phenotype exhibits dosage-sensitivity as animals heterozygous for P[*da*-gal4], P[UAS-Morgue] are fully viable while animals homozygous for one and heterozygous for the other P element exhibit extensive but generally incomplete lethality. This suggests that the lethal phenotype results only after a specific threshold level of Morgue expression is attained. This lethal phenotype provided a useful assay for Morgue activity and we utilized it to dissect the importance of the distinct domains of Morgue protein, as well as the conserved Gly421 active site residue. Analysis of Morgue deletion mutant proteins indicated that each domain contributes to Morgue-induced lethality and no single domain is absolutely essential. Morgue deletion mutants lacking the zinc finger or F Box were also able to enhance Grim-Reaper-induced eye cell death similarly to native Morgue. However, deletion of the UEV domain did weaken Morgue’s cell death-enhancing activities. These findings suggest that Morgue functions as an F box protein are not critical for cell death functions while the UEV domain is critical. This is consistent with Morgue being a multi-functional protein that may have distinct actions in different tissues. For example, Morgue may have multiple ubiquitination functions; it could have separable actions in an E2/UEV conjugase complex as well and SCF type ligase complex. Analysis of Morgue Gly421x point mutant proteins revealed that Alanine, Cysteine, or Serine substitutions at this position did not alter Morgue-induced lethality or cell death enhancement. Given the complete conservation of the active site Gly421 residue in the Morgue UEV domain, this result was unexpected and it differs from the phenotypic effects of Morgue Gly421x overexpression in the eye. It is possible that the UEV domain could have activities independent of the catalytic site residue or that the Gly421 residue is important for Morgue functions distinct from those that generate the over-expression phenotypes. In either case, the effects of ectopic gene expression may not completely or accurately reflect all the native functions of the endogenous gene.

### Morgue interacts with SkpA and polyubiquitin *in vivo*

The Morgue protein contains several potential protein interaction domains, suggesting that Morgue normally functions in conjunction with other factors. Previous yeast 2-hybrid and cell culture assays identified several potential Morgue-interacting proteins, including SkpA [35] as well as SkpC [46], both components of ubiquitin SCF-type E3 ligase complexes. Morgue was also shown to associate with SkpA via GST-pulldown assays and this interaction was dependent upon the presence of the Morgue F box [26]. Additional *in vitro* assays indicated that Morgue can associate with DIAP1 and may downregulate DIAP1 levels in dying cells [25], [26]. In order to identify the proteins that Morgue associates with *in vivo*, we utilized a P[*da*-Gal4], P[UAS-3xFlag:Morgue] strain and isolated Morgue-associated proteins from whole adult flies. This analysis identified Morgue association with three small proteins, each less than 30 kDa. Tryptic digest, mass spectrometry, and amino acid sequence analyses were used to identify these three proteins. Two of these, each ∼20 kDa, correspond to SkpA. This result is consistent with previous *in vitro* assays demonstrating Morgue/SkpA interaction and provides and strongly suggests that the *in vivo* interaction assay was effective in identifying bona fide Morgue interactors. This result also suggests that Morgue does function as an F box protein, either in an SCF-type E3 ligase complex or in a distinct capacity. However, Morgue actions as an F box protein are not essential for the over-expression phenotypes of lethality or cell death enhancement. This in turn suggests that Morgue has multiple distinct functions and may carry out different actions in different processes. For example, Morgue may act as an F box protein in circadian rhythms pathways but not in other processes such as cell death.

Morgue was also shown to associate with a ubiquitin multimer. The apparent size and peptide sequence of the ubiquitin multimer suggests that it corresponds to a dimer or trimer; Morgue also associates with ubiquitin tetramers *in vitro*. The presence of a diagnostic tryptic peptide indicates that the multimer has a branched stoichiometry with a Lys48 linkage. Determination of the steric configuration of the Morgue-associated ubiquitin chain provides some insight into the potential functions of Morgue. Thus, a Lys48 linkage indicates that Morgue functions in regulating the targeting of substrate proteins to the 26S proteasome 41, [42], a conclusion consistent with Morgue’s ability to influence DIAP1 protein levels [25], [26]. The result also indicates that Morgue does not associate with partially processed, linear ubiquitin moieties derived from a polyubiquitin precursor. Morgue and ubiquitin association was a somewhat surprising result. Other UEVs have been shown to bind ubiquitin monomers that are added to substrate protein or growing ubiquitin chains [6]-[9]. Morgue may act similarly to some E2s and E3 that act together as putative E4s to regulate ubiquitin chain elongation [46], [47]. However, the association of Morgue with a K48-linked ubiquitin multimer suggests that Morgue promotes addition of polyubiquitin chains onto substrate proteins. Alternately, as yeast 2-hybrid assays indicate that Morgue can associate with the de-ubiquitinating enzyme Ubp64e [35], Morgue could be involved in removing ubiquitin chains from substrate proteins. This would suggest that Morgue promotes deubiquitination and increased levels of substrate proteins. While this function does not to fit well with Morgue’s downregulation of DIAP1 levels, it has not yet been established that Morgue acts directly on DIAP1 ubiquitination. Morgue could rescue distinct pro-apoptotic or circadian rhythm factors from degradation via the proteasome. Furthermore, Morgue could regulate the ubiquitination status of substrate proteins via both addition and removal of ubiquitin moieties.

### Morgue is a Novel Type of Ubiquitin-binding Protein

Interestingly, Morgue association with polyubiquitin was mediated not only via the COOH-terminal region of Morgue that contains the UEV domain, but also through the NH_2_-terminal region containing the zinc finger and F box. This suggests that multiple, distinct regions of Morgue are capable of binding ubiquitin. UEV domains correspond to ubiquitin-binding motifs and have been shown to bind ubiquitin monomers. They possess several residues present in enzymatically active E2s that are important for Ub interaction, including the critical Asparagine77 [4], [6], [48], [49]. In the case of yeast MMS2, non-covalent association with ubiquitin helps position ubiquitin for attachment and use by the associated Ubc13 E2 conjugase [6], [8], [9]. However, binding of UEVs to ubiquitin multimers has not yet been described. It is possible that the unique Gly421 residue in the E2 active site of Morgue facilitates Morgue association with ubiquitin multimers. Other ubiquitin-binding structures include zinc finger domains, such as the NZF, UBP and A20 domains [20]-[22]. While the Morgue zinc finger does not correspond to any previously identified ubiquitin-binding zinc finger type, it is similar in organization to the ubiquitin-binding C4 zinc finger motif of Npl4 [50]. This suggests that the Morgue zinc finger may constitute a novel ubiquitin-binding domain. However, it is not yet clear that the Morgue zinc finger is actually responsible for polyubiquitin-binding and it may be that a distinct domain in the Morgue NH_2_ region is required. The only other conserved domain in the NH_2_-region is the F box. It would seem unlikely that the F box mediates polyubiquitin binding as F box functions have been well analyzed and F box/ubiquitin association has not been described. Nonetheless, the F box and zinc finger could function cooperatively to bind polyubuiquitin. Overall, the Morgue protein could be bifunctional where two distinct regions of Morgue are involved in binding to polyubiquitin. These regions could separately and simultaneously associate with a distinct ubiquitin multimer, either for distinct or common functions. Alternately, these regions could act cooperatively to associate with a ubiquitin multimer. Taken together, the results of our *in vivo* and *in vitro* association assays suggest that Morgue defines a novel, distinct type of ubiquitin-binding protein containing multiple ubiquitin-binding domains.

## Acknowledgments

The authors wish to thank Kathy Matthews and the Bloomington *Drosophila* Stock Center for supplying us with *Drosophila* strains and N. Perrimon for providing pUASt vector DNA. We are also grateful to J. Wakefield for supplying us with anti-SkpA serum. We thank Dr. Ming Li for critical reading of the manuscript.

## Supporting Information

Figure S1 **Mass spectrometry analysis of Morgue-associated protein sample 1.** Note several major peaks released from tryptic digestion of Morgue-associated proteins, including prominent peak at MW 1460.8002 daltons (red circle). Y-axis = % intensity of peaks, X-axis = mass.
(PDF)

## References

[1] Glickman MH, Ciechanover A (2002) The ubiquitin-proteasome proteolytic pathway: destruction for the sake of construction. Physiol Rev 82: 373–428. 11917093 10.1152/physrev.00027.2001

[2] Pickart CM, Eddins MJ (2004) Ubiquitin: structures, functions, mechanisms. Biochim Biophys Acta 1695: 55–72. 15571809 10.1016/j.bbamcr.2004.09.019

[3] Ciechanover A (2005) Intracellular protein degradation: from a vague idea thru the lysosome and the ubiquitin-proteasome system and onto human. diseases and drug targeting. Cell Death Differ 12: 1178–1190. 16094394 10.1038/sj.cdd.4401692

[4] VanDemark AP, Hofmann RM, Tsui C, Pickart CM, Wolberger C (2001) Molecular insights into polyubiquitin chain assembly: crystal structure of the Mms2/Ubc13 heterodimer. Cell 105: 711–720. 11440714 10.1016/s0092-8674(01)00387-7

[5] Tsui C, Raguraj A, Pickart CM (2005) Ubiquitin binding site of the ubiquitin E2 variant (UEV) protein Mms2 is required for DNA damage tolerance in the yeast RAD6 pathway. J Biol Chem 280: 19829–19835. 15772086 10.1074/jbc.M414060200

[6] Eddins MJ, Carlile CM, Gomez KM, Pickart CM, Wolberger C (2006) Mms2-Ubc13 covalently bound to ubiquitin reveals the structural basis of linkage-specific polyubiquitin chain formation. Nat Struct Mol Biol 13: 915–920. 16980971 10.1038/nsmb1148

[7] Hau DD, Lewis MJ, Saltibus LF, Pastushok L, Xiao W, et al (2006) Structure and interactions of the ubiquitin-conjugating enzyme variant human Uev1a: Implications for enzymatic synthesis of polyubiquitin chains. Biochem 45: 9866–9877. 16893187 10.1021/bi060631r

[8] Lewis MJ, Saltibus LF, Hau DD, Xiao W, Spyracopoulos L (2006) Structural basis for non-covalent interaction between ubiquitin and the ubiquitin conjugating enzyme variant human MMS2. J Biomol NMR 34: 89–100. 16518696 10.1007/s10858-005-5583-6

[9] Patushok L, Spyracopoulos L, Xiao W (2007) Two Mms2 residues cooperatively interact with ubiquitin and are critical for Lys63 polyubiquitination in vitro and in vivo. FEBS Lett 581: 5343–5348. 17964296 10.1016/j.febslet.2007.10.028

[10] Hofmann RM, Pickart CM (1999) Noncanonical MMS2-encoded ubiquitin- conjugating enzyme functions in assembly of novel polyubiquitin chains for DNA repair. Cell 96: 645–653. 10089880 10.1016/s0092-8674(00)80575-9

[11] Garrus JE, von Schwedler UK, Pornillos OW, Morham SG, Zavitz KH, et al (2001) Tsg101 and the vacuolar protein sorting pathway are essential for HIV-1 budding. Cell 107: 55–65. 11595185 10.1016/s0092-8674(01)00506-2

[12] Sundquist WI, Schubert HL, Kelly BN, Hill GC, Holton JM, et al (2004) Ubiquitin recognition by the human TSG101 protein. Mol Cell 13: 783–739. 15053872 10.1016/s1097-2765(04)00129-7

[13] Willems AR, Schwab M, Tyers M (2004) A hitchhiker’s guide to the cullin ubiquitin ligases: SCF and its kin. Biochimica et Biophys Acta 1695: 133–170. 10.1016/j.bbamcr.2004.09.027 15571813

[14] Deshaies RJ, Joazeiro CA (2009) RING domain E3 ubiquitin ligases. Ann Rev Biochem 78: 399–434. 19489725 10.1146/annurev.biochem.78.101807.093809

[15] Nagy V, Dikic I (2010) Ubiquitin ligase complexes: from substrate selectivity to conjugational specificity. Biol Chem 39: 163–169. 10.1515/bc.2010.021 20030582

[16] VanDemark AP, Hill CP (2002) Structural basis of ubiquitylation. Curr Opin Struct Biol 12: 822–830. 12504688 10.1016/s0959-440x(02)00389-5

[17] Pickart CM, Fushman D (2004) Polyubiquitin chains: polymeric protein signals. Curr Opin Chem Biol 8: 610–616. 15556404 10.1016/j.cbpa.2004.09.009

[18] Hicke L (2001) Protein regulation by monoubiquitin. Nat Rev Mol Cell Biol 2: 195–201. 11265249 10.1038/35056583

[19] Hicke L, Dunn R (2003) Regulation of membrane protein transport by ubiquitin and ubiquitin-binding proteins. Ann Rev Cell Dev Biol 19: 141–172. 14570567 10.1146/annurev.cellbio.19.110701.154617

[20] Hurley JH, Lee S, Prag G (2006) Ubiquitin-binding domains. Biochem J 1399: 361–372. 10.1042/BJ20061138 PMC1615911 17034365

[21] Dikic I, Wakatsuki S, Walters KJ (2009) Ubiquitin-binding domains – from structures to functions. Nat Rev Mol Cell Biol 10: 659–671. 19773779 10.1038/nrm2767 PMC7359374

[22] Husnjak K, Dikic I (2012) Ubiquitin-binding proteins: Decoders of ubiquitin-mediated cellular functions. Ann Rev Biochem 81: 291–322. 22482907 10.1146/annurev-biochem-051810-094654

[23] Kerscher O, Felberbaum R, Hochstrasser M (2006) Modification of proteins by ubiquitin and ubiquitn-like proteins. Ann Rev Cell Dev Biol 22: 159–180. 16753028 10.1146/annurev.cellbio.22.010605.093503

[24] Denuc A, Marfany G (2010) SUMO and ubiquitin paths converge. Biochem Soc Trans 38: 34–39. 20074031 10.1042/BST0380034

[25] Hays R, Wickline L, Cagan R (2002) Morgue mediates apoptosis in the Drosophila melanogaster retina by promoting degradation of DIAP1. Nat Cell Biol 4: 425–431. 12021768 10.1038/ncb794

[26] Wing JP, Schreader BA, Yokokura T, Wang Y, Andrews PS, et al (2002) Drosophila Morgue is an F box/ubiquitin conjugase domain protein important for grim-reaper mediated apoptosis. Nat Cell Biol 4: 451–456. 12021772 10.1038/ncb800

[27] Schreader BA, Wang Y, Carter S, Grigas J, Nambu JR (2010) Drosophila morgue influences cell numbers and positions in the embryonic nervous system. Int J Dev Biol 54: 1425–1433. 21302253 10.1387/ijdb.092979bs

[28] Murad A, Emery-Le M, Emery P (2007) A subset of dorsal neurons modulates circadian behavior and light responses in Drosophila. Neuron 53: 689–701. 17329209 10.1016/j.neuron.2007.01.034 PMC1852515

[29] Schreader BA, Wang Y, Nambu JR (2003) Drosophila morgue and the intersection between protein ubiquitination and programmed cell death. Apoptosis 8: 129–139. 12766473 10.1023/a:1022914524601

[30] Zhou Y, Carpenter ZW, Brennan GT, Nambu JR (2009) The unique Morgue protein is conserved in a diverse but restricted set of invertebrates. Mol Biol Evol 26: 2245–2259. 19602541 10.1093/molbev/msp147 PMC2766937

[31] Brand AH, Perrimon N (1993) Targeted gene expression as a means of altering cell fates and generating dominant phenotypes. Development 118: 401–415. 8223268 10.1242/dev.118.2.401

[32] Patel NH (1994) Imaging neuronal subsets and other cell types in whole-mount Drosophila embryos and larvae using antibody probes. In: Drosophila melanogaster: practical uses in cell and molecular biology, methods in cell. biology, vol. 44 (Goldstein LSB, Fryberg EA, editors), 446–485. New York: Academic Press. 10.1016/s0091-679x(08)60927-9 7707967

[33] Mohseni N, McMillan SC, Chaudhary R, Mok J, Reed BH (2009) Autophagy promotes caspase-dependent cell death during Drosophila development. Autophagy 5: 329–338. 19066463 10.4161/auto.5.3.7444

[34] Vaessin H, Brand M, Jan LY, Jan YN (1994) daughterless is essential for neuronal precursor differentiation but not for initiation of neuronal precursor formation in Drosophila embryo. Development 120: 935–945. 7600969 10.1242/dev.120.4.935

[35] Giot L, Bader JS, Brouwer C, Chaudhuri A, Kuang B, et al (2003) A protein interaction map of Drosophila. Science 302: 1727–1736. 14605208 10.1126/science.1090289

[36] Arribas C, Sampedro J, Izquierdo M (1986) The ubiquitin genes in D. melanogaster: transcription and polymorphism. Biochim Biophys Acta 868: 119–127.

[37] Lee HS, Simon JA, Lis JT (1988) Structure and expression of ubiquitin genes of Drosophila melanogaster. Mol Cell Biol 8: 4727–4735. 2463465 10.1128/mcb.8.11.4727-4735.1988 PMC365564

[38] Turner GC, Varshavsky A (2000) Detecting and measuring cotranslational protein degradation in vivo. Science 289: 2117–2220. 11000112 10.1126/science.289.5487.2117

[39] Peng J, Schwartz D, Elias JE, Thoreen CC, Cheng D, et al (2003) A proteomics approach to understanding protein ubiquitination. Nat Biotechnol 21: 921–926. 12872131 10.1038/nbt849

[40] Kaiser P, Wohlschlegel J (2005) Identification of ubiquitination sites and determination of ubiquitin-chain architectures by mass spectrometry. Methods Enzymol 3999: 266–277. 10.1016/S0076-6879(05)99018-6 16338362

[41] Ikeda F, Dikic I (2008) Atypical ubiquitin chains: new molecular signals. EMBO Reports 9: 536–542. 18516089 10.1038/embor.2008.93 PMC2427391

[42] Trempe JF (2011) Reading the ubiquitin postal code. Curr Opin Struct Biol 21: 792–801. 22036065 10.1016/j.sbi.2011.09.009

[43] Kirisako T, Kamei K, Murata S, Kato M, Fukumoto H, et al (2006) A ubiquitin ligase complex assembles linear polyubiquitin chains. EMBO J 25: 4877–4887. 17006537 10.1038/sj.emboj.7601360 PMC1618115

[44] Sinha SK, Brew K (1981) A label selection selection procedure for determining the location of protein-protein interaction sites by cross-linking with bismidoesters. Application to lactose synthase. J Biol Chem 256: 4193–4204. 6783656

[45] Guruharsha KG, Rual JF, Zhai B, Mintseris J, Vaidya P, et al (2011) A protein complex network of Drosophila melanogaster. Cell 147: 690–703. 22036573 10.1016/j.cell.2011.08.047 PMC3319048

[46] Ye Y, Rape M (2009) Building ubiquitin chains: E2 enzymes at work. Nat Rev Mol Cel Biol 10: 755–764. 10.1038/nrm2780 PMC3107738 19851334

[47] Metzger MB, Weissman AM (2010) Working on a chain: E3s ganging up for ubiquitylation. Nat Cell Biol 12: 1124–1126. 21124306 10.1038/ncb1210-1124

[48] Lin D, Tatham MH, Yu B, Kim S, Hay RT, et al (2002) Identification of a substrate recognition site on Ubc9. J Biol Chem 277: 21740–21748. 11877416 10.1074/jbc.M108418200

[49] Ozkan E, Yu H, Deisenhofer J (2005) Mechanistic insight into the allosteric activation of ubiquitin-conjugating enzyme by RING-type ubiquitin ligases. Proc Natl Acad Sci U S A 102: 18890–18895. 16365295 10.1073/pnas.0509418102 PMC1316884

[50] Wang B, Alam SL, Meyer HH, Payne M, Stemmler TL, et al (2003) Structure and ubiquitin interactions of the conserved zinc finger domain of Npl4. J Biol Chem 278: 20225–20234. 12644454 10.1074/jbc.M300459200 PMC3366119

