# Supplementary figures and images for: Drosophila Morgue Associates with SkpA and Polyubiquitin In Vivo
Source: PLoS One. 2013 Sep 30;8(9):e74860. doi: 10.1371/journal.pone.0074860 (PMC3787007; doi:10.1371/journal.pone.0074860)

Supplemental Figure 1.

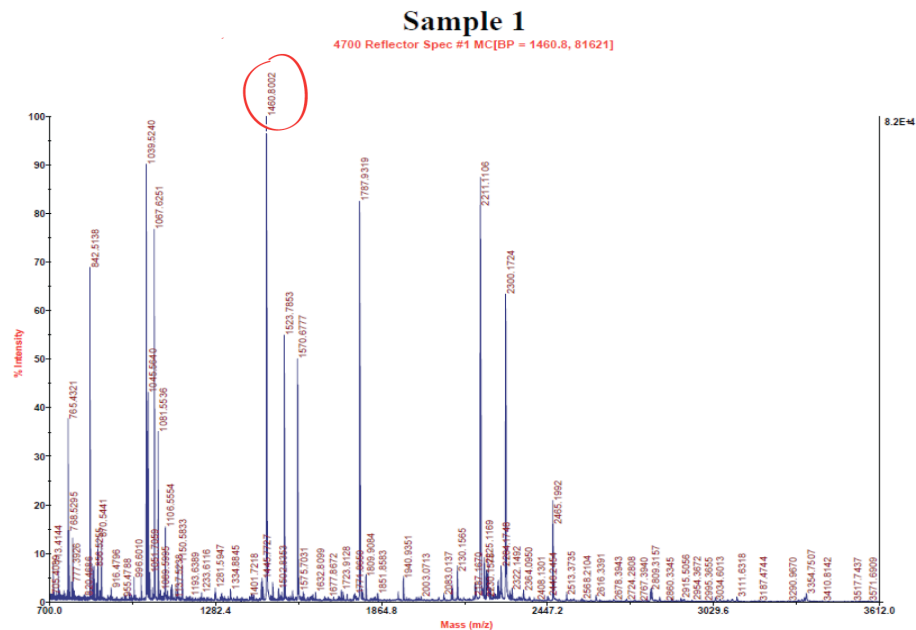

Supplement: Figure S1 — Mass spectrometry analysis of Morgue-associated protein sample 1. Note several major peaks released from tryptic digestion of Morgue-associated proteins, including prominent peak at MW 1460.8002 daltons (red circle). Y-axis = % intensity of peaks, X-axis = mass. (PDF) [file pone.0074860.s001.pdf]
